# Calloused hands, shorter life? Occupation and older-age survival in Mexico

**DOI:** 10.4054/demres.2020.42.32

**Published:** 2020-05-19

**Authors:** Hiram Beltrán-Sánchez, Noreen Goldman, Anne R. Pebley, Josefina Flores Morales

**Affiliations:** 1 Fielding School of Public Health, California Center for Population Research, University of California at Los Angeles, USA.; 2 Office of Population Research, Princeton University, USA.; 3 Fielding School of Public Health, California Center for Population Research, University of California at Los Angeles, USA.; 4 Department of Sociology, California Center for Population Research, University of California at Los Angeles, USA.

## Abstract

**BACKGROUND:**

Inequalities in mortality are often attributed to socioeconomic differences in education level, income, and wealth. Low socioeconomic status (SES) is generally related to worse health and survival across the life course. Yet, disadvantaged people are also more likely to hold jobs requiring heavy physical labor, repetitive movement, ergonomic strain, and safety hazards.

**OBJECTIVE:**

We examine the link between primary lifetime occupation, together with education and net worth, on survival among older adults in Mexico.

**METHODS:**

We use data from four waves (2001, 2003, 2012, and 2015) of the Mexican Health and Aging Study (MHAS). We estimate age-specific mortality rates for ages 50 and over using a hazards model based on a two-parameter Gompertz function.

**RESULTS:**

Primary lifetime occupations have a stronger association with survival for women than men. Women with higher socioeconomic status have significantly lower mortality rates than lower status women, whether SES is assessed in terms of schooling, wealth, or occupation. Occupational categories are not jointly related to survival among men, even without controls for education and wealth. There are significant survival differences by wealth among men, but no disparities in mortality by education.

**CONCLUSIONS:**

Consistent with recent studies of the Mexican population, we fail to find the expected gradient in the association between some measures of SES and better survival among men.

**CONTRIBUTION:**

Our estimates extend this anomalous pattern among Mexican men to another dimension of SES, occupation. SES differentials in mortality are substantially larger for Mexican women, highlighting an important gender disparity.

## 1. Introduction

In most populations, conventional measures of socioeconomic status (SES) – education level, income, and wealth – are associated with a broad range of health outcomes. Higher SES is generally related to better health and survival across the life course. This effect diminishes with age, but remains important for older adults (Smith and Goldman 2007). Although these relationships have been most frequently identified in wealthy countries, the growing body of research in middle-income countries generally confirms these findings despite some indication that the associations are more erratic and weaker than in higher-income settings (Goldman et al. 2011). However, what has been far less studied overall is the linkage between work and health (Ahonen et al. 2018; Landsbergis et al. 2018), particularly in middle-income countries where research on occupational health is relatively recent. In particular, few studies focus on the effects of physically or psychologically difficult work, which varies considerably both across and within socioeconomic groups, on longer-term health. The failure to incorporate lifetime working conditions in research on health inequalities has occurred despite longstanding recognition of occupation both as an important component of SES and in the history of research on improving occupational health.

Undoubtedly, a substantial part of the influence of work on health operates through conventional SES measures. For example, schooling is a primary determinant of occupational attainment and individuals’ jobs are strongly related to their income and wealth throughout the life course. Nevertheless, job characteristics and working conditions are likely to have impacts on health not fully captured by conventional SES measures. These effects may affect health through a diverse set of pathways, including physical conditions and/or hazardous exposure, adverse psychosocial stressors such as heavy job demands and lack of control, or the types of social networks and connections associated with jobs (Burgard and Lin 2013; Clougherty, Souza, and Cullen 2010; Toch et al. 2014). These effects are likely to be stronger in middle-income than in wealthy countries, given the frequent lack of occupational safety and health regulations in the former contexts (Beltrán-Sánchez, Pebley, and Goldman 2017).

Our objective in this paper is to examine the link between primary lifetime occupation and a well-measured and important health outcome – survival – in Mexico. Mexico is a particularly interesting setting for this analysis: while it has experienced part of the transition to a service and information economy common in higher-income countries, its manufacturing sector has also grown, and it has a sizeable percentage of jobs in agricultural and other extractive industries (e.g., oil). Thus, physically (and potentially psychologically) difficult jobs remain relatively common. This is particularly true because development and enforcement of occupational health and safety regulations are relatively recent and weaker than in many wealthier countries (Sánchez-Román et al. 2006). Mexico shares with Chile the highest income inequality ranking in the world according to the Organization for Economic Cooperation and Development (OECD 2019).

The role of work in the life course in Mexico can be quite different for men and women. While work, marriage, and providing for a family are major tasks in men’s lives, marriage and motherhood are generally seen as central to women’s lives (Fussell 2005; Parrado and Zenteno 2001), although this traditional gender division of labor is changing (García and de Oliveira 2001). From the 1950s to the 1970s, Mexican women had low rates of labor force participation and high fertility rates. Most women confined their work to homemaking and raising children, as long as family finances permitted (García and de Oliveira 2001). By contrast, virtually all Mexican men were in the labor force from before or right after leaving school. Since the 1970s, Mexican men’s labor force participation rates and timing of life course transitions have changed relatively little (except to accommodate later school leaving), but women’s lives have changed substantially (Fussell 2005; García, and de Oliveira 2001). In particular, total fertility rates markedly declined (from 6.6 children per woman in 1970 to 2.6 in 2000) (United Nations 2019), and women’s labor force participation significantly increased (Parrado and Zenteno 2001). For example, Parrado and Zenteno (2001) show that 44.7% of women born between 1936 and 1938 had ever worked by age 30. The comparable figure for women born between 1951 and 1953 was 52.4%, and for women born between 1966 and 1968 was 66.3%. Examining the effect of these changes on Mexican women’s life course transitions, Fussell (2005) shows that there has been relatively little change in ages at marriage and first birth. Instead, women increasingly combine employment with family roles and childbearing at younger ages, and then increase the amount they work later in life. Mexican women often cycle in and out of employment, especially at younger ages (Fussell 2005).

Mexican women’s likelihood of employment varies by family socioeconomic status: higher-status women are more able than middle-class women to combine traditional family roles and employment by hiring domestic help (Parrado and Zenteno 2001), while for women from lower-income families, work may be an important component of family economic survival strategies, particularly during the frequent economic crises that Mexico has experienced since the 1980s (Fussell 2005; García and de Oliveira 2001). Housework and childcare themselves may be physically strenuous, regardless of whether they are done in one’s own home or elsewhere for pay. Whether women who are out of the labor market do their own housework and childcare also depends on socioeconomic status, since it is common for higher-status women, regardless of outside employment, to hire household help.

All of these factors complicate analysis of the effects of work on women’s long-term health and survival. For this reason and because of the substantial differences in the role of employment and work in the life course of Mexican men and women, our results differentiate between men and women throughout the analysis.

To date, little research has examined linkages between occupation and all-cause mortality in Mexico (Haro-García et al. 2013; Sánchez-Román et al. 2006). However, a recent analysis based on national survey data in Mexico examines the relationship between adults’ primary lifetime occupation and mobility limitations (Beltrán-Sánchez, Pebley, and Goldman 2017). The study demonstrates that primary occupation contributes to socioeconomic differentials in mobility limitations at older ages: coefficients for educational attainment and wealth were reduced in size when job categories were included in statistical models. In addition, job categories generally retained their significant association with mobility limitations even when education and wealth were controlled for. Given strong evidence across diverse settings that the prevalence of mobility limitations is one of the strongest predictors of survival (Goldman, Glei, and Weinstein 2016), there is every reason to expect a significant association between primary lifetime occupation and older-age mortality in Mexico.

However, two recent papers using national data for Mexico suggest that it may be an exception to the pervasive positive association between higher levels of SES and increased survival. Both papers find unexpected patterns between education and survival for men, i.e., either no gradient or a reverse gradient (Rosero-Bixby 2018; Saenz and Wong 2016). The authors speculate that one explanation may be selective mortality of the most vulnerable and frail individuals, e.g., a process that would result in greater loss by the older ages of the lowest-education groups and those born in earlier periods when mortality risks were high and health-related resources were scarce. To the extent that these strong selection processes have been operative in Mexico, mortality differences by occupation may also be minimal or nonexistent.

In this paper we use data from the nationally representative Mexican Health and Aging Study (MHAS) to examine the importance of occupation for socioeconomic inequalities in survival. We begin the analysis by examining the consistency of estimated death rates for adults ages 50+ in the MHAS and external data sources. We then examine the associations between survival and three indicators of SES – education, wealth, and primary lifetime occupation – to assess the gross effects of each of these measures on survival along with the net effects of occupation. A particular interest is not only to examine the degree of variation in older-adult mortality among primary occupations but also to assess the similarity of findings between men and women. An earlier study finds greater variation in mobility limitations across occupations for women than men, a result which may be due at least in part to much higher rates of mobility limitations across the life course for women (Beltrán-Sánchez, Pebley, and Goldman 2017). An important question is whether this sex difference applies to survival, an outcome where women have a distinct advantage over men.

## 2. Data and methods

Data for this analysis come from four waves (2001, 2003, 2012, and 2015) of the Mexican Health and Aging Study (MHAS), a longitudinal study of older Mexicans (Mexican Health and Aging Study 2001). The first wave in 2001 was based on a nationally representative sample of Mexicans who were born prior to 1951 and lived in Mexico. In this analysis all data on covariates comes from the 2001 survey, whereas data on survival status come from subsequent waves. The sample includes residents of all 32 Mexican states and of urban and rural areas (Wong, Espinoza, and Palloni 2007; Wong, Michaels-Obregon, and Palloni 2015). If the respondent was married or in a consensual union and the spouse/partner lived in the household, the spouse/partner was also interviewed regardless of age. Interviews were conducted in person, with proxy interviews in cases of poor health, hospitalization, and temporary absence. We restricted the analysis to those 50 and older who were interviewed in the 2001 survey without proxy respondents (proxies did not answer questions on net worth). The individual response rate was 91.8% at baseline (Wong, Espinoza, and Palloni 2007; Wong, Michaels-Obregon, and Palloni 2015). We excluded the few respondents with missing values on the variables of interest: primary lifetime job (n = 6), education (n = 8), and net worth (n = 19). The final analytic sample at baseline comprises 12,419 respondents, 5,690 men and 6,729 women (see Appendix A).

### 2.1 Explanatory variables

#### 2.1.1 Socioeconomic status

The analysis is based on three SES variables that were reported by the survey respondent at baseline in 2001: primary lifetime occupation, education, and net worth. We use the occupation associated with each respondent’s primary lifetime job reported at the baseline interview in response to the following: “For the following questions, please think about the activities that you performed in your main job throughout your life. What is the name of the job, profession, post, or position you held in your main job?” ^5^ (Appendix B). Textual responses to these questions were coded by the MHAS project using the Mexican occupation classification system created by the Mexican National Institute of Statistics, Geography, and Informatics (INEGI). These occupations are not comparable to the international standard classification of occupations created by the International Labor Organization.

We would ideally like to have data indicating the physical and psychological work conditions each respondent experienced in his/her main job, but unfortunately this information does not exist either in MHAS or in other sources for Mexico. Instead, we used job categories as proxies for exposure to heavy physical work demand. We focused on physically demanding jobs because they are more apparent from job titles and classifications than psychologically stressful jobs. We created a set of categories that reflect the likely physical work demands of jobs, based on the limited data available on Mexican occupational health and safety (Sánchez-Román et al. 2006) and knowledge of the Occupational Information Network (O*NET) in the US (https://www.onetonline.org/). These are based on the 19 groups in the original INEGI classification corresponding to the “major categories” in the list of occupations, including one group for respondents with no occupation at the time of interview (see Appendix C1). We then combined several jobs with a low level of physical strain into a single category to yield the 15 occupational groups for this analysis (see Appendix C2). For example, we combined “Officials and Directors in the Public, Private, and Social Sectors” with “Department Heads, Coordinators, and Supervisors in Administrative and Service Activities.” We also grouped a few other categories of jobs which, based on the limited information available for Mexico and data from the US, are likely to have similar physical work demands (e.g., “Operators of Fixed Machinery and Equipment for Industrial Production” with “Drivers and Assistant Drivers of Mobile Machinery and Transport Vehicles”). Combining occupational groups into a smaller number of categories also ensured that we had adequate cell sizes for the analysis. Since many Mexican women spend much of their adult life as homemakers, we considered grouping unemployed women in the “domestic” occupation category. However, as described above, there is substantial variation in the activities of women who are not in the labor market, e.g., many women in higher-income households hire household and childcare help. Thus, we assigned those not reporting any outside employment to a separate “never worked” category.

Consistent with our discussion in the introduction, 67.9% of women reported ever working compared to 99.5% for men. Substantial gender segregation in occupation is also apparent: the occupational distribution varies markedly between men and women, as shown in Table 1. The most common job category for women who reported outside work is domestic work; the most common category for men is agriculture, livestock, forestry and fishing – reflecting the time period in which these respondents were likely to have held their primary jobs (i.e., the 1970s through the 1990s).

We examine occupation differentials together with two conventional SES measures reported by the respondents: education and wealth (net worth). Educational attainment is typically completed by early adulthood, thereby mitigating potential problems of reverse causality. We classify education into three categories: no schooling, 1–6 years (primary school), and 7+ years (more than completed primary). The MHAS measure of net worth is based on the monetary value of all assets (including businesses, land, housing, stocks and bonds, savings, etc.) minus debts for individuals (or for the couple if the respondent was married/cohabiting). The MHAS project estimated wealth values while imputing missing values in the components of wealth with the method of sequence regressions (see Wong, Espinoza, and Palloni 2007). The imputation method has several advantages: allowing variable values to be zero, accounting for other imputed variables, and incorporating responses based on categorical responses (‘unfolding brackets’). We use three categories of wealth based on deciles of the distribution (deciles 1 to 5, 6 to 9, and 10). This parsimonious categorization provides a non-linear association in which we can assess mortality differentials between respondents in the top 10% and in the 60%–90% of the distribution relative to the lower half of the net worth distribution.

#### 2.1.2 Outcome

The baseline sample was followed for 14 years through the subsequent three waves (2003, 2012, and 2015) to ascertain vital status and last time of contact. Appendices D1 and D2 document the attrition of the sample through death, non-response, and loss-to-follow-up. The outcome variable in this analysis is mortality, derived from interviews with a family member or someone who had information about the respondent. These interviews include questions on age at death and month and year of death. There were 3,804 reported deaths between 2001 and 2015 in our analytic sample.

**Figure 1:**
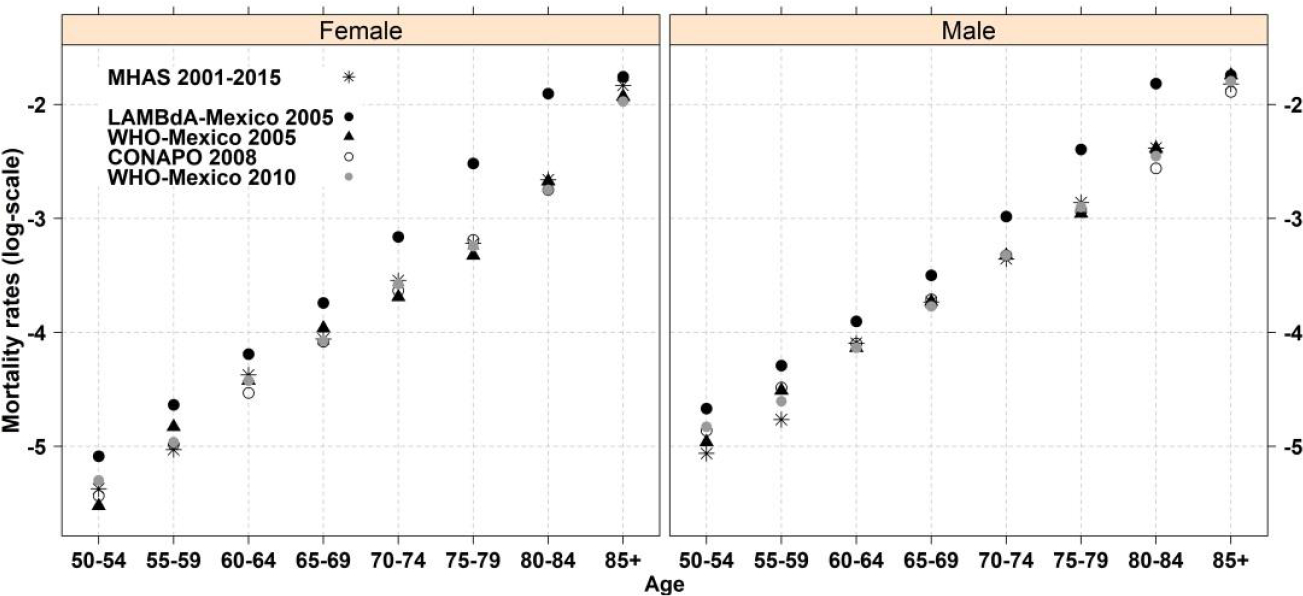
Age-specific mortality rates (per person-year) by sex in MHAS 2001–2015 and comparisons with other sources *Notes*: CONAPO denotes the Mexican Population Council (Consejo Nacional de Poblacion), WHO denotes the World Health Organization and LAMBdA denotes the Latin American Mortality Database (https://www.ssc.wisc.edu/cdha/latinmortality/).

Figure 1 compares five-year age-specific mortality rates (log scale) by sex from the MHAS for the period 2001–2015 with corresponding rates obtained from the Latin American Mortality Database (LAMBdA) (Palloni, Pinto, and Beltrán-Sánchez 2014), the Mexican Population Council (CONAPO), which is the institution in Mexico that produces official mortality estimates for the country (Partida Bush and García Guerrero 2018), and the World Health Organization (WHO) (World Health Organization 2018). See Appendix E for further details. The WHO estimates come directly from the Mexican official death registration system, which Palloni et al. (2014) find to be subject to age misstatement at older ages. The LAMBdA estimates have been adjusted for age misstatement (i.e., the systematic overstatement of ages in either population or deaths), which is a type of error that affects observed age distributions in many countries (see, for example, Coale and Kisker 1986 and Condran, Himes, and Preston 1991) but is particularly salient and acute in Latin America (Palloni, Pinto, and Beltrán-Sánchez 2014). The figure shows that the MHAS mortality rates for each sex are very similar to those from the WHO and to the official rates from CONAPO. However, they differ from values in LAMBdA, especially for ages 70 to 84 and for women. The fact that the MHAS rates for each sex are very similar to those from CONAPO and the WHO suggests that MHAS mortality rates at older ages may also be affected by age overstatement. Unfortunately, there is no way to correct data for age misstatement of individual MHAS respondents. However, this problem is likely to be diminished in our analysis because, as described later, our focus is on relative rather than absolute death rates and because respondents’ ages are obtained at first interview.

### 2.2 Analytic strategy

We estimate age-specific mortality rates for ages 50 and over using a hazards model based on a two-parameter Gompertz function, with time measured by age, using Stata v15.1 (StataCorp. 2017) and the R software (R Core Team 2019). Models are estimated for the total population as well as by sex. Estimates of age are obtained from reported dates of birth in months, collected in the baseline interview.

At early stages of the analysis we estimated a series of non-proportional hazard models, for each sex and for the total population, in which age was interacted with net worth and with education, thereby permitting the effects of these SES measures on mortality to vary by age (Appendix F). Because the interaction terms were jointly significant (Wald test at p<0.05) in only one of 18 models – about the same as would occur by chance – we present results from proportional hazard models. In the final analysis we estimate six proportional hazard models for each sex and present the results as hazard ratios; these hazard ratios are risks of dying for individuals in a particular category relative to those in the reference (omitted) group. To assess gross effects of each of the three SES variables, we include them one at a time in the model (models (1), (2), and (4)). To assess net effects of the occupation variables we consider models that include education and net worth, one at a time and together, in models that also include the 15 occupational categories (models (3), (5), and (6)).

## 3. Results

Summary statistics for the explanatory variables are presented in Appendix Table A-1. The male sample is slightly older than the female sample, a reversal of the typical age differential because of the inclusion of spouses in the sample (i.e., male spouses of sampled women of a given age are considerably older than female spouses of sampled men of the same age). Men have, on average, one more year of schooling than women (5 vs. 4 years) and about a quarter (24.2%) have more than an elementary school education (7+ years) versus one-fifth among women (19.7%).

Table 1 presents estimates of life expectancy at age 50 (e_50_ or LE50) by sex for each occupational group based on age-specific hazard models that include the 15 job categories, without the other SES measures. The values indicate substantial variation in e_50_ across occupations, ranging from 27.1 to 40.0 for women and from 26.5 to 31.3 for men. Not surprisingly given women’s higher life expectancy, estimates of e_50_ are as large as or, more often, larger for women than men in each occupation. For women, the occupation associated with the lowest e_50_ is assistant laborers in industrial production, repair, and maintenance; this occupation has the next-to-lowest value of e_50_ for men, with educators having the lowest value. The occupation with the highest e_50_ is professionals, for both men and women.

These job differentials in e_50_ are reflected in the hazard ratios shown in Table 2 for women and Table 3 for men (see Appendix F for a complete table of results). Estimates from model (1) in Table 2, which examines the gross effects of occupational groups, indicate that, among women, the occupational categories are jointly significant (p ≅ 0.02), with more than half of the occupations having significantly higher mortality than professionals. Hazard ratios for several occupations are notable. For example, the mortality risks for the category of assistants and laborers in industrial production are almost four times as high as those of professionals, while for the three job groups of traveling salespeople; technicians; and agriculture, livestock, forestry, and fishing the corresponding relative risks are approximately threefold. Relative risks are also high (about 2.5) for domestic workers – the second largest occupational group for women (after no occupation, which also has a high relative risk of about 2.7). As described in the introduction, selection into professional occupations is undoubtedly higher for women than for men, yet female differentials remain large in comparison to other reference groups. For example, the mortality risks for assistants and laborers in industrial production are more than double those of directors in various sectors (2.2). The lowest-risk jobs for women, apart from professionals, are directors in various sectors, operators of machinery and equipment, administrative support staff, and educators.

The hazard ratios in model (1) for men (Table 3) are much smaller than those for women. More importantly, in contrast to the finding for women, the occupational categories for men are not jointly significant at p<0.05.

Estimates for models (2) and (4) indicate significant disparities in mortality for women by both education and net worth (Table 2). Women with at least seven years of education have significantly better survival than those without schooling and the likelihood of survival increases with higher levels of wealth. However, the corresponding estimates for men (Table 3) reveal virtually no difference by years of schooling. The net worth variables are significant for men in the expected direction, although, once again, the hazard ratios are considerably closer to 1 than those for women.

The addition of education and/or net worth variables to models including the job categories (models (3), (5), and (6)) only slightly reduces the hazard ratios for men’s occupations (Table 3), although none of the three jobs with significant gross effects for men have significant net effects in model (6). By contrast, the reduction in the gross effects for women’s occupations with inclusion of the additional SES variables is substantial and the job categories are no longer jointly significant in model (6); as evidenced by the estimates in models (3) and (5), both education and wealth contribute to these reductions. This result is not surprising, given that the education and wealth differentials are much larger for women than for men. However, although the importance of primary lifetime occupation for women’s survival is clearly driven in part by disparities in schooling and wealth, two occupations continue to be associated with significantly and substantially elevated mortality risks in the final model (assistants and laborers in industrial production and travelling sales people).

## 4. Discussion

The primary goal of this analysis has been to examine whether jobs are an important source of social inequality in survival and whether this relationship varies by sex. It appears that jobs have a much stronger association with survival for women than men, with or without controls for education and wealth. These findings mirror those from an earlier study that determines that the associations between job categories and mobility limitations are considerably stronger for women than men (Beltrán-Sánchez, Pebley, and Goldman 2017).

Beltrán-Sánchez, Pebley, and Goldman (2017) find that the occupations associated with the largest number of mobility limitations are generally those that are likely to involve poor physical work conditions, such as frequent injuries, heavy physical demands, or repetitive positions or movements. To a large extent, we find that the same types of jobs are also associated with the highest death rates. However, with the inclusion of education and wealth variables, only two job categories (both for women) have significantly elevated mortality rates compared to the lowest risk group of professionals.

Like two previous studies, we fail to find the expected gradient of the association between higher levels of schooling and better survival among men. Both of the earlier studies are based on MHAS samples, although with somewhat different inclusion criteria, control variables, time periods, and modeling strategies than our analysis. Rosero-Bixby (2018) finds a strong reverse gradient in life expectancy at age 60 (e_60_) during the period 2002–2012 by years of schooling for men, i.e., lower life expectancy with increased education (as well as a modest reverse gradient between wealth and e_60_ for men). During a similar time period (2001–2012) as the Rosero-Bixby study, Saenz and Wong (2016) stratify their analysis by cohort (but not by sex) and demonstrate that education is not significantly associated with mortality for the cohort born before 1940. The authors offer several hypotheses for these findings, including selective survival of the most robust individuals during periods of high mortality risk, low prevalence of obesity among older cohorts, and faulty data, but they lack the information needed to refute or support them.

Buttenheim and colleagues (Buttenheim et al. 2010a; Buttenheim et al. 2010b) explore another plausible argument for the absence of male SES gradients. They maintain that higher levels of SES are not necessarily protective against detrimental health behaviors, particularly for countries at lower levels of development or during periods of rapid economic change; in these circumstances, wealthier and more-educated individuals may have better access to cigarettes and high-calorie diets – a pattern which would attenuate SES differences in survival. Based on the 2000 Mexican National Health Survey (ENSA), they find that SES gradients in smoking behavior and obesity are complex, often demonstrating reverse or no differentials by educational attainment or household wealth. However, these anomalous gradients are apparent for both sexes, thus failing to provide support for the sex difference documented in our analysis. The inadequacy of explanations focused on health-related behaviors is reinforced by Hummer et al. (2014), who analyze MHAS data – which is restricted to older individuals, in contrast to the broad adult age range in ENSA – for a more recent period (2012) than the 2000 ENSA study. They find only weak education differentials for men and women in a broad set of cardiovascular risk factors that includes smoking and obesity.

Although our results are distinct from those of Rosero-Bixby (2018) – we find no gradient rather than a reverse gradient in survival by education among men – our estimates underscore the absence of the education differential in survival – which is generally found in higher-income countries. Moreover, our analysis extends this anomalous pattern among Mexican men to another dimension of SES, occupation. The occupational categories are not jointly related to survival among men, even without controls for education and wealth. This result is particularly surprising because (1) all three SES measures are significantly associated with mobility limitations among Mexican men in an earlier study (Beltrán-Sánchez, Pebley, and Goldman 2017); and (2) the number of mobility limitations is one of the strongest predictors of older-adult mortality in many populations (Goldman, Glei, and Weinstein 2016). A potentially related puzzle characterizes Mexicans residing in the United States: the Mexican-origin population lives longer than non-Latino whites despite their lower SES – a phenomenon often referred to as the ‘Latino Paradox’ – yet Latinos of Mexican origin, and Latinos more generally, have substantially higher rates of functional limitations (e.g., mobility limitations) and disability than non-Latino whites (Haas and Rohlfsen 2010; Hayward et al. 2014; Markides et al. 2007). Scholars have speculated that compared with other ethnic groups the relatively high rates of functional impairment and disability among Latinos may result more from the physically demanding and dangerous types of occupations that Latinos hold and less from chronic diseases (Goldman 2016; Hayward et al. 2014). Thus, the link between functional limitations and survival may be weaker for Latinos than for other groups in the United States, a relationship that may also be true for men residing in Mexico.

Consistent with the significant differences in life course between Mexican men and women and the fact that women’s survival rates are almost universally higher than men’s, the findings for women differ substantially from those for men: women with higher SES have significantly lower mortality rates than lower-status women, whether SES is assessed in terms of schooling, wealth, or type of occupation. One plausible explanation of differences between social gradients in mortality for men and women is that higher education and professional and managerial employment are more common for men than for women in Mexico. Because women who achieve higher education and professional or managerial careers are more highly selected on a broad range of characteristics, the health status differential between professional and non-professional women may be greater than the comparable differences for men. It is also possible that women at the bottom of the occupational ladder, in jobs such as laborers and technicians, may do more hazardous tasks, work longer hours, and experience harsher conditions, or may be more subject to serious injury and physical limitations, relative to men in the same occupations. Lower-income women may also be more likely to have a ‘double shift’ – i.e., difficult work at their job plus the work and responsibility of caring for a family and household outside of the job.

Given the broad age range of our sample, we assessed the robustness of our results to restricting the sample to respondents aged 50–65 at baseline (see Appendix G). As in the full sample, differentials by net worth are significant for men, whereas differences in schooling are not. The wealth differentials are slightly larger in this younger sample compared with the full sample of men. For women, the differentials by both schooling and net worth are larger in the younger sample and are significant. These comparisons suggest that SES differentials in survival may have increased for younger cohorts or that selective mortality may have weakened the gradient in the sample that includes older respondents. The increase in the education gradient for women may also result from the greater number of years of schooling within a given educational category for women aged 50–65 compared with older women.

There are several limitations to the present analysis. As with many social science surveys, the most vulnerable groups (e.g., the homeless or persons in institutions) are likely to be under-represented in the sample. Moreover, the MHAS includes only a single question about the respondent’s main job, rather than an occupational history. Richer data that reflects multiple jobs over the life course might reveal stronger associations with survival. The use of broad job categories in this analysis and the reliance on proxy reports of survival rather than vital registration data may also contribute to attenuation of the estimates. There is also comparatively little information about other aspects of women’s and men’s life course transitions or about the activities of respondents who report never having been employed. This lack is particularly problematic for interpreting the results for women, many of whom remain outside the paid work force or cycle in and out of it to accommodate family needs.

There are two potential reliability problems in the data. The first is age misstatement in the MHAS. We suspect that the structure of the data we use – in which respondents report their own age at the first wave in 2001 when they are 50+ years old and are then followed as they age – is likely to mitigate the age misstatement problem. Moreover, although age misstatement biases the reported age-specific death rates, our analysis relies on relative rather than absolute death rates. If age is more likely to be overstated among those in low SES groups, deaths would appear to be occurring at older ages for these groups, on average, than they really are. In this case, our analysis would provide an underestimate of SES differentials in mortality.

The second potential problem is omission of deaths of respondents in the sample due to attrition. Note that the MHAS continues to locate respondents for two consecutive follow-up interviews before they can be considered lost-to-follow-up. Any deaths that are missing are classified in the “lost-to-follow-up” category. Fortunately, the number in that category is relatively small, especially given the 14-year follow-up period (see Appendix D2). For example, between baseline and follow-up in 2015 about 4.7% of respondents were lost-to-follow-up with unknown vital status (see Appendices D1 and D2). Another related problem is differential omission of deaths by SES, which might occur, for example, if the MHAS had a harder time re-contacting people of low SES in subsequent interviews. In fact, the opposite pattern may have occurred: a smaller fraction of people with low SES and unknown vital status might have been lost-to-follow-up than with high SES. Thus, to the extent that lower SES individuals are less likely than their higher SES counterparts to be reported as dead, our results may overestimate the magnitude of the SES differentials. Nevertheless, previous research by Rosero-Bixby (2018) demonstrates that wide-ranging assumptions regarding the relative mortality risk of those lost-to-follow-up in the MHAS compared with survey respondents have only a modest effect on mortality differentials by wealth and education (see Appendix to Rosero-Bixby (2018) at: https://www.demographic-research.org/volumes/vol38/3/files/demographic-research.38-3.zip).

Of considerable importance is the fact that we are unable to make any causal statements about the linkages between occupation and survival from these data. Nevertheless, this analysis raises two intriguing questions that merit additional research. One relates to the anomalous gradients in survival found among Mexican men for both education and occupation, in contrast to patterns found for mobility limitations. The second is why the associations between occupation and survival are much stronger among Mexican women than Mexican men.

## Supplementary Material

Appendices

## Figures and Tables

**Table 1:** Sample distribution by the final set of job categories at baseline, total number of deaths at follow-up, and life expectancy at age 50: MHAS 2001‒2015

		Total population			Female			Male		
Job category	code	N	Deaths	LE50	N	Deaths	LE50	N	Deaths	LE50

No occupation		2,182	691	30.3	2,154	678	30.5	28	13	NA
Professional	110–119	247	38	33.0	57	5	40.0	190	33	31.3
Technicians	120–129	298	75	29.0	168	38	30.1	130	37	27.7
Educators	130–139	366	83	30.5	232	46	32.8	134	37	26.5
Directors in the public, private, and social sectors, department heads, coordinators, and supervisors in administrative and service activities	210–219, 610–619	356	83	30.4	90	15	34.3	266	68	29.2
Agriculture, livestock, forestry, and fishing	410–419	2,026	780	29.6	452	154	30.2	1,574	626	29.3
Bosses, supervisors, etc. in artistic and industrial production and in repair and maintenance activities, artisans and workers in production, repair, and maintenance	510–519, 520–529	1,932	615	29.2	610	180	31.2	1,322	435	28.3
Operators of fixed machinery and equipment for industrial production, drivers and assistant drivers of mobile machinery and transport vehicles	530–539, 550–559	745	235	28.3	98	20	33.2	647	215	27.5
Assistants, laborers, etc. in industrial production, repair, and maintenance	540–549	292	104	27.2	119	37	27.1	173	67	27.1
Administrative support staff	620–629	559	112	31.8	384	60	33.3	175	52	29.5
Merchants and sales representatives	710–719	1,076	256	30.4	676	150	31.7	400	106	28.2
Traveling salespeople and traveling salespeople of services	720–729	244	76	28.7	142	40	29.0	102	36	28.3
Workers in the service industry, safety and security personnel	810–819, 830–839	713	228	29.2	302	77	30.8	411	151	28.0
Domestic workers	820	1,264	384	30.9	1,227	372	31.0	37	12	NA
Other workers	140–149, 990–992	119	44	27.6	19	5	NA	100	39	27.6

TOTAL		12,419	3,804		6,730	1,877		5,689	1,927	

*Notes*: Life expectancy (LE) estimated from Gompertz hazards models fitted separately for the total population, men, and women, with time measured by age. LE50 is estimated for occupations with at least 50 respondents. NA indicates occupations with fewer than 50 respondents.

**Table 2:** Hazard ratio estimates from Gompertz survival models for the MHAS sample 2001‒2015 for females

		Gross effect										Net effect	
		
Variables		(1) job		(2) educ		(3) educ+job		(4) nworth		(5) nworth+job		(6) educ+nworth+job	
	code	HR	95% C.I.	HR	95% C.I.	HR	95% C.l.	HR	95% C.I.	HR	95% C.I.	HR	95% C.I.

Age		1.10	[1.10–1.11]	1.10	[1.10–1.11]	1.10	[1.10–1.11]	1.10	[1.10–1.10]	1.10	[1.09–1.10]	1.10	[1.09–1.10]
Occupation categories (ref=Professional)													
No occupation		2.68	[1.11–6.47]			2.40	[0.99–5.81]			2.30	[0.95–5.55]	2.14	[0.88–5.20]
Technicians	120–129	2.80	[1.10–7.13]			2.70	[1.06–6.87]			2.56	[1.01–6.51]	2.51	[0.99–6.38]
Educators	130–139	2.09	[0.83–5.27]			2.08	[0.83–5.24]			1.93	[0.77–4.87]	1.94	[0.77–4.88]
Directors in the public, private, and social sectors, department heads, coordinators, and supervisors in administrative and service activities	210–219, 610–619	1.79	[0.65–4.93]			1.75	[0.64–4.81]			1.80	[0.65–4.94]	1.76	[0.64–4.85]
Agriculture, livestock, forestry, and fishing	410–419	2.76	[1.13–6.73]			2.41	[0.98–5.92]			2.31	[0.95–5.65]	2.13	[0.87–5.24]
Bosses, supervisors, etc. in artistic and industrial production and in repair and maintenance activities, artisans and workers in production, repair, and maintenance	510–519, 520–529	2.48	[1.02–6.03]			2.22	[0.91–5.42]			2.09	[0.86–5.11]	1.96	[0.80–4.79]
Operators of fixed machinery and equipment for industrial production, drivers and assistant drivers of mobile machinery and transport vehicles	530–539, 550–559	2.00	[0.75–5.34]			1.83	[0.68–4.88]			1.73	[0.65–4.63]	1.64	[0.61–4.38]
Assistants, laborers, etc. in industrial production, repair, and maintenance	540–549	3.89	[1.53–9.90]			3.44	[1.35–8.81]			3.30	[1.29–8.41]	3.06	[1.19–7.84]
Administrative support staff	620–629	1.99	[0.80–4.95]			1.96	[0.79–4.87]			1.77	[0.71–4.41]	1.76	[0.71–4.39]
Merchants and sales representatives	710–719	2.35	[0.96–5.73]			2.16	[0.88–5.27]			2.06	[0.84–5.02]	1.95	[0.80–4.78]
Traveling salespeople and traveling salespeople of services	720–729	3.15	[1.24–7.98]			2.87	[1.13–7.28]			2.71	[1.07–6.88]	2.56	[1.01–6.52]
Workers in the service industry, safety and security personnel	810–819, 830–839	2.59	[1.05–6.41]			2.33	[0.94–5.78]			2.20	[0.89–5.45]	2.06	[0.83–5.12]
Domestic workers	820	2.53	[1.05–6.12]			2.22	[0.91–5.41]			2.09	[0.86–5.07]	1.93	[0.79–4.71]
Other workers	140–149, 990–992	NA	NA			NA	NA			NA	NA	NA	NA
Education categories (ref=No schooling)													
1–6 years of schooling				1.00	[0.90–1.11]	1.00	[0.90–1.12]					1.02	[0.92–1.14]
7+ years of schooling				0.82	[0.73–0.92]	0.87	[0.76–0.99]					0.92	[0.80–1.05]
Net worth (ref=deciles 1–5)													
Deciles 6–9								0.82	[0.74–0.90]	0.82	[0.75–0.91]	0.83	[0.75–0.91]
Decile 10								0.64	[0.53–0.77]	0.67	[0.55–0.81]	0.68	[0.56–0.83]

Gamma		0.0964		0.0954		0.0957		0.0953		0.0952		0.0949	
Observations		6,730		6,730		6,730		6,730		6,730		6,730	

Wald tests (p-values)													
Occupation		0.0182				0.118				0.0684		0.131	
Education				0.0012:	2	0.0608						0.252	
Net worth								1.27e-07		1.92e-06		7.41 e-06	

AIC		8889		8880		8888		8861		8866		8867	
BIC		8998		8907		9010		8888		8989		9003	

*Notes*: NA indicates occupations with fewer than 50 people. AIC denotes Akaike Information Criterion and BIC denotes Bayesian Information Criterion.

**Table 3:** Hazard ratio estimates from Gompertz survival models for the MHAS sample 2001‒2015 for males

		Gross effect										Net effect	
		
Variables		(1) job		(2) educ		(3) educ+job		(4) nworth		(5) nworth+job		(6) educ+nworth+job	
	code	HR	95% C.I.	HR	95% C.I.	HR	95% C.I.	HR	95% C.I.	HR	95% C.I.	HR	95% C.I.

Age		1.09	[1.09–1.10]	1.09	[1.09–1.10]	1.09	[1.09–1.10]	1.09	[1.09–1.10]	1.09	[1.09–1.10]	1.09	[1.09–1.10]
Occupation categories (ref=Professional)													
No occupation		NA	NA			NA	NA			NA	NA	NA	NA
Technicians	120–129	1.44	[0.90–2.31]			1.44	[0.90–2.30]			1.39	[0.87–2.24]	1.39	[0.87–2.24]
Educators	130–139	1.63	[1.02–2.61]			1.63	[1.02–2.61]			1.59	[0.99–2.55]	1.59	[0.99–2.56]
Directors in the public, private, and social sectors, department heads, coordinators, and supervisors in administrative and service activities	210–219, 610–619	1.23	[0.81–1.87]			1.23	[0.81–1.86]			1.21	[0.80–1.84]	1.21	[0.80–1.84]
Agriculture, livestock, forestry, and fishing	410–419	1.23	[0.87–1.75]			1.18	[0.82–1.69]			1.15	[0.80–1.65]	1.12	[0.77–1.63]
Bosses, supervisors, etc. in artistic and industrial production and in repair and maintenance activities, artisans and workers in production, repair, and maintenance	510–519, 520–529	1.36	[0.96–1.94]			1.33	[0.92–1.90]			1.28	[0.89–1.84]	1.26	[0.87–1.83]
Operators of fixed machinery and equipment for industrial production, drivers and assistant drivers of mobile machinery and transport vehicles	530–539, 550–559	1.47	[1.02–2.12]			1.44	[0.99–2.09]			1.38	[0.95–2.01]	1.37	[0.94–2.00]
Assistants, laborers, etc. in industrial production, repair, and maintenance	540–549	1.53	[1.01–2.33]			1.47	[0.96–2.26]			1.43	[0.93–2.19]	1.40	[0.91–2.16]
Administrative support staff	620–629	1.20	[0.77–1.85]			1.19	[0.77–1.84]			1.14	[0.73–1.77]	1.13	[0.73–1.77]
Merchants and sales representatives	710–719	1.37	[0.93–2.03]			1.35	[0.91–1.99]			1.33	[0.89–1.97]	1.31	[0.88–1.96]
Traveling salespeople and traveling salespeople of services	720–729	1.35	[0.84–2.17]			1.30	[0.80–2.10]			1.26	[0.78–2.04]	1.23	[0.76–2.01]
Workers in the service industry, safety and security personnel	810–819, 830–839	1.40	[0.96–2.04]			1.36	[0.93–1.99]			1.31	[0.89–1.93]	1.29	[0.87–1.91]
Domestic workers	820	NA	NA			NA	NA			NA	NA	NA	NA
Other workers	140–149, 990–992	1.47	[0.92–2.33]			1.43	[0.89–2.28]			1.37	[0.85–2.20]	1.35	[0.84–2.18]
Education categories (ref=No schooling)													
1–6 years of schooling				0.98	[0.88–1.10]	0.96	[0.86–1.07]					0.97	[0.86–1.08]
7+ years of schooling				0.97	[0.86–1.09]	0.93	[0.81–1.06]					0.95	[0.83–1.09]
Net worth (ref=deciles 1–5)													
Deciles 6–9								0.89	[0.81–0.98]	0.88	[0.80–0.97]	0.89	[0.80–0.98]
Decile 10								0.85	[0.72–1.01]	0.87	[0.73–1.03]	0.87	[0.73–1.04]

Gamma		0.0887		0.0874		0.0881		0.0874		0.0885		0.0881	
Observations		5,689		5,689		5,689		5,689		5,689		5,689	

Wald tests (p-values)													
Occupation		0.400				0.337				0.416		0.383	
Education				0.862		0.555						0.763	
Net worth								0.0199		0.0241		0.0330	

AIC		8760		8750		8762		8743		8756		8760	
BIC		8866		8777		8882		8769		8876		8892	

*Notes*: NA indicates occupations with fewer than 50 people. AIC denotes Akaike Information Criterion and BIC denotes Bayesian Information Criterion.

## Footnotes

5 The original wording in Spanish is as follows: “Para las siguientes preguntas, por favor piense acerca de las actividades que realizó en su trabajo principal a lo largo de su vida. ¿Cuál es el nombre del oficio, profesión, puesto o cargo que desempeña en su trabajo principal?”
