# Supplementary material for: Calloused hands, shorter life? Occupation and older-age survival in Mexico
Source: Demogr Res. Author manuscript; Available in PMC 2023 Feb 10. (PMC9917739; doi:10.4054/demres.2020.42.32)
Supplement: Appendices [file NIHMS1825198-supplement-Appendices.docx]

**Appendix A:** **Sample selection (MHAS-Wave I: 2001)**

Section H contains information on functional limitations and section I on occupation.

Where:

I.1 Have you ever had a job for which you received a payment or profit?

I.2 Have you ever helped in a business, farm, or ranch without receiving payment or profit?

I.9 What is the name of the office, profession, or place where you worked in your main job?

Respondents with no occupation, I.1=NO & I.2=NO (non-working):

| Sex | N | % |
| --- | --- | --- |
| Male | 30 | 1.4 |
| Female | 2,159 | 98.6 |
| Total | 2,189 | 100.0 |

Table A1. Sample characteristics of MHAS respondents, Wave I (unweighted)

| Characteristic | Total | Male | Female |
| --- | --- | --- | --- |
|  | N=12,419 | N=5,689 | N=6,730 |
| Age (mean) | 62.1 | 62.3 | 61.9 |
| Years of education |  |  |  |
| Mean | 4.49 | 5.01 | 4.06 |
| Education categories (%) |  |  |  |
| 0 years | 24.9 | 21.9 | 27.4 |
| 1-6 years | 53.4 | 53.9 | 52.9 |
| 7+ years | 21.7 | 24.2 | 19.7 |
| Net worth (Mexican pesos*) |  |  |  |
| Mean | 388,500 | 422,800 | 359,400 |
| Deciles (range) |  |  |  |
| D1 | (-585000 ; 5308) | (-279663 ; 13000) | (-585000 ; 2000) |
| D2 | (5308 ; 46781) | (13000 ; 55000) | (2000 ; 37000) |
| D3 | (46781 ; 91000) | (55000 ; 100000) | (37000 ; 82287) |
| D4 | (91000 ; 148296) | (100000 ; 156821) | (82287 ; 131423) |
| D5 | (148296 ; 206139) | (156821 ; 219211) | (131423 ; 200000) |
| D6 | (206139 ; 286000) | (219211 ; 300000) | (200000 ; 273797) |
| D7 | (286000 ; 396660) | (300000 ; 413918) | (273797 ; 377154) |
| D8 | (396660 ; 543960) | (413918 ; 576702) | (377154 ; 515000) |
| D9 | (543960 ; 855926) | (576702 ; 903541) | (515000 ; 822861) |
| D10 | (855926 ; 26127040) | (903541 ; 26127040) | (822861 ; 26127040) |

* 1 dollar corresponds to about 19 Mexican pesos as of July 18, 2019.

Note: net worth represents the value of all assets (including businesses, land, housing, stocks and bonds, savings, etc.) ***minus*** debts for individuals or for the couple if married/cohabiting.

**Appendix B: Coding of primary occupation at baseline**

Occupation is based on question i.9 about “main job throughout life (primary occupation)” in MHAS

Note: Textual responses to these questions were coded by the MHAS project using the Mexican occupation classification system created by the Mexican National Institute of Statistics, Geography and Informatics (INEGI). Thus, the corresponding variables in the MHAS dataset only contain occupational codes. Appendix C1 shows these codes and their associated occupations.

**Appendix C1: Primary occupation categories at baseline and number of deaths as of 2015**

**Step 1**: Original INEGI classification of job categories using 19 main groups: sample size (N) and number of deaths for the total population and by sex

|  |  |  | Total | |  | Female | |  | Male | |
| --- | --- | --- | --- | --- | --- | --- | --- | --- | --- | --- |
| Job category | | code | N | Deaths |  | N | Deaths |  | N | Deaths |
| 0 | No occupation |  | 2,182 | 691 |  | 2,154 | 678 |  | 28 | 13 |
| 1 | Professional | 110-119 | 247 | 38 |  | 57 | 5 |  | 190 | 33 |
| 2 | Technicians | 120-129 | 298 | 75 |  | 168 | 38 |  | 130 | 37 |
| 3 | Educators | 130-139 | 366 | 83 |  | 232 | 46 |  | 134 | 37 |
| 4 | Workers in Art, Shows, and Sports | 140-149 | 60 | 20 |  | 4 | 1 |  | 56 | 19 |
| 5 | Directors in the Public, Private, and Social Sectors | 210-219 | 183 | 42 |  | 48 | 7 |  | 135 | 35 |
| 6 | Agriculture, Livestock, Forestry, and Fishing | 410-419 | 2,026 | 780 |  | 452 | 154 |  | 1,574 | 626 |
| 7 | Bosses, Supervisors, etc. in Artistic and Industrial Production and in Repair and Maintenance Activities | 510-519 | 119 | 37 |  | 15 | 2 |  | 104 | 35 |
| 8 | Artisans and Workers in Production, Repair, and Maintenance | 520-529 | 1,813 | 578 |  | 595 | 178 |  | 1,218 | 400 |
| 9 | Operators of Fixed Machinery and Equipment for Industrial Production | 530-539 | 294 | 89 |  | 96 | 18 |  | 198 | 71 |
| 10 | Assistants, Laborers, etc. in Industrial Production, Repair, and Maintenance | 540-549 | 292 | 104 |  | 119 | 37 |  | 173 | 67 |
| 11 | Drivers and Assistant Drivers of Mobile Machinery and Transport Vehicles | 550-559 | 451 | 146 |  | 2 | 2 |  | 449 | 144 |
| 12 | Department Heads, Coordinators, and Supervisors in Administrative and Service Activities | 610-619 | 173 | 41 |  | 42 | 8 |  | 131 | 33 |
| 13 | Administrative Support Staff | 620-629 | 559 | 112 |  | 384 | 60 |  | 175 | 52 |
| 14 | Merchants and Sales Representatives | 710-719 | 1,076 | 256 |  | 676 | 150 |  | 400 | 106 |
| 15 | Traveling Salespeople and Traveling Salespeople of Services | 720-729 | 244 | 76 |  | 142 | 40 |  | 102 | 36 |
| 16 | Workers in the Service Industry | 810-819 | 532 | 164 |  | 292 | 76 |  | 240 | 88 |
| 17 | Domestic Workers | 820 | 1,264 | 384 |  | 1,227 | 372 |  | 37 | 12 |
| 18 | Safety and Security Personnel | 830-839 | 181 | 64 |  | 10 | 1 |  | 171 | 63 |
| 19 | Other Workers | 990-992 | 59 | 24 |  | 15 | 4 |  | 44 | 20 |
|  | TOTAL |  | 12,419 | 3,804 |  | 6,730 | 1,877 |  | 5,689 | 1,927 |

**Appendix C2: Reduced primary occupation categories at baseline**

We combine some occupation categories to increase the sample size. The combined categories are shown above in similar color with a full description below.

| Occupations in the Original INEGI classification | Final occupation categories shown in the table below |
| --- | --- |
| 0. No occupation | 1. No occupation |
| 1. Professional (110-119) | 2. Professional (110-119) |
| 2. Technicians (120-129) | 3. Technicians (120-129) |
| 3. Educators (130-139) | 4. Educators (130-139) |
| 5. Officials and Directors in the Public, Private, and Social Sectors (210-219)  12. Department Heads, Coordinators, and Supervisors in Administrative and Service Activities (610-619) | 5. Officials and Directors in the Public, Private, and Social Sectors, Department Heads, Coordinators, and Supervisors in Administrative and Service Activities (210-219, 610-619) |
| 6. Agriculture, Livestock, Forestry, and Fishing (410-419) | 6. Agriculture, Livestock, Forestry, and Fishing (410-419) |
| 7. Bosses, Supervisors, etc. (510-519)  8. Artisans and Workers in Production, Repair, and Maintenance (520-529) | 7. Bosses, Supervisors, etc. in Artistic and Industrial Production and in Repair and Maintenance Activities, Artisans and Workers in Production, Repair, and Maintenance (510-519, 520-529) |
| 9. Operators of Fixed Machinery and Equipment for Industrial Production (530-539)  11. Drivers and Assistant Drivers of Mobile Machinery and Transport Vehicles (550-559) | 8. Operators of Fixed Machinery and Equipment for Industrial Production, Drivers and Assistant Drivers of Mobile Machinery and Transport Vehicles (530-539, 550-559) |
| 10. Assistants, Laborers, etc. in Industrial Production, Repair, and Maintenance (540-549) | 9. Assistants, Laborers, etc. in Industrial Production, Repair, and Maintenance (540-549) |
| 13. Administrative Support Staff (620-629) | 10. Administrative Support Staff (620-629) |
| 14. Merchants and Sales Representatives (710-719) | 11. Merchants and Sales Representatives (710-719) |
| 15. Traveling Salespeople and Traveling Salespeople of Services (720-729) | 12. Traveling Salespeople and Traveling Salespeople of Services (720-729) |
| 16. Workers in the Service Industry (810-819)  18. Safety and Security Personnel (830-839) | 13. Workers in the Service Industry, Safety and Security Personnel (810-819, 830-839) |
| 17. Domestic Workers (820) | 14. Domestic Workers (820) |
| 4. Workers in Art, Shows, and Sports (140-149)  19. Other Workers (990-992) | 15. Other workers (140-149, 990-992) |

**Appendix D1: Follow up sample from baseline with total sample size in each wave, MHAS Waves I to IV: 2001-2015.**

Note: “Proxy” corresponds to interviews conducted by someone other than the respondent in cases of poor health, hospitalization, and temporary absence. “Not eligible” corresponds to spouses/partners who were interviewed at some point but who are not part of the sampling design (i.e., they are not the targets). “LFU” denotes lost to follow-up and “Non-resp” denotes non-response.

The coding scheme for each of the categories shown above is based on two questions in each follow up survey, “Type of interview (tipent_X)” and “Type of non-interview (tipne_X)” using the following codes:

|  |  | 2003 | | 2012 | | 2015 | |
| --- | --- | --- | --- | --- | --- | --- | --- |
| Category | Acronym | tipent_03 | tipne_03 | tipent_12 | tipne_12 | tipent_15 | tipne_15 |
| 1.Interview: complete | 1.Interview | 11,12 |  | 1,2 |  | 1,2 |  |
| 2.Interview: Proxy | 2.Proxy | 21,22 |  | 3,4 |  | 3,4 |  |
| 3.Interview: non-response | 3.Non-resp | 42 |  |  |  |  |  |
| 4. Non-interview: incomplete | 4.Incomplete |  |  |  | 2,3,6 |  | 2,3,6 |
| 5. Non-interview: refused | 5.Refused |  | 22,23 |  | 8,9,10 |  | 8,9 |
| 6. Non-interview: LFU | 6.LFU |  | 21,25,26,29 |  | 4,5,12-19 |  | 4,5,10-17 |
| 7. Non-interview: known dead | 7.LFU-dead |  | 24,27 |  | 7,11,20 |  | 7 |
| 8.Dead | 8.Dead | 31,32,33 |  | 5 |  | 5 |  |
| 9. Not elegible | 9.Not elegible | elegible_03==0 | | elegible_12==0 | | elegible_15==0 | |

The next table shows the specific codes associated with each variable in each wave using the same color-scheme as shown above:

| **2003** | |  | **2012** | |  | **2015** | |
| --- | --- | --- | --- | --- | --- | --- | --- |
|  | **Type of interview 2003** tipent_03 |  |  | **Type of interview 2012** tipent_12 |  |  | **Type of interview 2015** tipent_15 |
| 11 | Direct, first interview |  | 1 | 1.Direct, follow-up interview |  | 1 | 1.Direct, follow-up interview |
| 12 | Direct, second interview |  | 2 | 2.Direct, new sample interview |  | 2 | 2.Direct, new spouse interview |
| 21 | Proxy, first interview |  | 3 | 3.Proxy, follow-up interview |  | 3 | 3.Proxy, follow-up interview |
| 22 | Proxy, second interview |  | 4 | 4.Proxy, new sample interview |  | 4 | 4.Proxy, new spouse interview |
| 31 | Next-of-kin interview, first interview |  | 5 | 5.Next-of-kin |  | 5 | 5.Next-of-kin |
| 32 | Next-of-kin interview, second interview |  |  |  |  |  |  |
| 33 | Next-of-kin interview, third interview |  |  |  |  |  |  |
| 42 | Non-response, second interview |  |  |  |  |  |  |
|  |  |  |  |  |  |  |  |
| **2003** | |  | **2012** | |  | **2015** | |
|  | **Type of non-interview 2003** tipne_03 |  |  | **Type of non-interview 2012**  tipne_12 |  |  | **Type of non-interview 2015**  tipne_15 |
| 0 | Interviewed |  | 1 | 1 Interviewed |  | 1 | 1.Complete Interview |
| 21 | Changed location and cannot be located |  | 2 | 2 Incomplete Interview |  | 2 | 2.Incomplete Interview |
| 22 | Refused Core Interview |  | 3 | 3 Postponed Interview |  | 3 | 3.Postponed Interview |
| 23 | Refused Proxy Interview |  | 4 | 4 Absent and there is no informant |  | 4 | 4.Absence of the subject or the proxy o |
| 24 | Refused Next of Kin Interview |  | 5 | 5 Empty household or informant not adec |  | 5 | 5.Absence of occupants or adequate info |
| 25 | Unable without informant |  | 6 | 6 Subject unable to complete interview |  | 6 | 6.Subject not fit for interview and withdraw |
| 26 | Absent and there is no informant |  | 7 | 7 Deceased without informant |  | 7 | 7.Subject deceased without next-of-kin |
| 27 | Deceased without an informant |  | 8 | 8 Refused |  | 8 | 8.Refusal |
| 29 | Other |  | 9 | 9 Refused direct interview |  | 9 | 9.Refusal from the subject or the proxy |
|  |  |  | 10 | 10 Refused proxy interview |  | 10 | 10.Change of residence |
|  |  |  | 11 | 11 Refused next-of-kin interview |  | 11 | 11.Subject could not be located |
|  |  |  | 12 | 12 Changed location |  | 12 | 12.Empty residence |
|  |  |  | 13 | 13 Subject could not be located |  | 13 | 13.Dwelling for temporary use |
|  |  |  | 14 | 14 Empty housing |  | 14 | 14.Dwelling with non-residential use, d |
|  |  |  | 15 | 15 Housing with temporary use |  | 15 | 15.Address not located |
|  |  |  | 16 | 16 Housing with non-residential use, de |  | 16 | 16.Area not safe |
|  |  |  | 17 | 17 Address not located |  | 17 | 17.Other |
|  |  |  | 18 | 18 Area not safe |  |  |  |
|  |  |  | 19 | 19 Other |  |  |  |
|  |  |  | 20 | 20 Deceased subject from the new sample |  |  |  |

**Appendix D2: Follow up sample from baseline (MHAS Waves I to IV: 2001-2015)**

The fraction of respondents lost to follow up (LFU) with unknown vital status is higher among people with high vs. low SES:


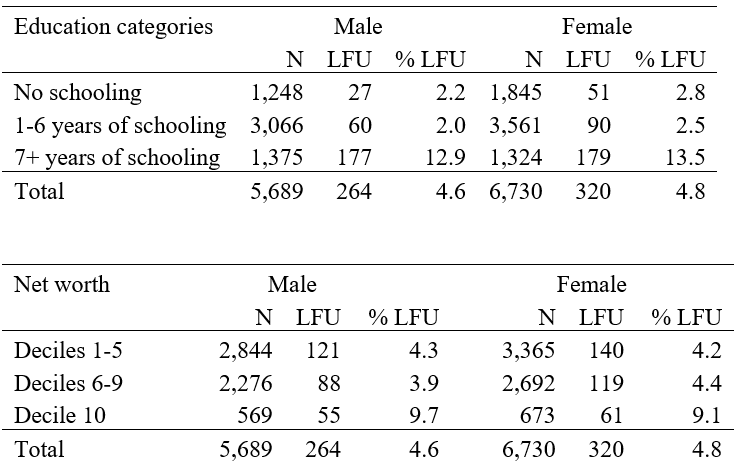


Between baseline and follow up in 2015 there were 584 respondents (264 males and 320 females corresponding to 4.7% of the baseline sample) with unknown vital status (335 respondents lost to follow up (LFU) —missing in two consecutive follow ups, and 249 partially lost to follow up—have not been located once, between 2012 and 2015).

Note: “missing” corresponds to respondents with missing information on both the type of interview and the type of non-interview.

**Appendix E: Age-specific mortality rates in MHAS 2001-2015 and comparisons with other sources**

**Deaths reported by next-of-kin.** The total number of deaths is shown in Table D3.1; note that these deaths are those that occurred among the baseline sample of 12,419 (see Appendix A for how the sample was selected). About 95% of deaths that occurred by 2003 and by 2012 have a complete date of death (month and year) with a negligible fraction of deaths occurring by 2015 having a missing date of death (1%) (Panel A). There is a higher fraction of deaths with missing date of death among women than men (Panel B). We imputed missing dates of death by assuming a uniform distribution between the follow-up years and/or the months of a year.

Table D3.1. Date of death (month and year) by follow-up wave.

We then estimated age-specific mortality rates for the MHAS. Figure 1 shows age-specific mortality rates for women and men, respectively, and comparisons with other data sources (The Latin American Mortality Database (LAMBdA) and life tables for Mexico from the World Health Organization in 2005 and 2010). Results indicate lower mortality rates in MHAS, particularly at ages 70+, relative to estimates for Mexico 2005 in LAMBdA, but similar results between MHAS and WHO. There are larger discrepancies between MHAS and LAMBdA among women.

Using the crude mortality rates we estimate life expectancy at age 50, e_50_, in MHAS (see Table E1). Overall, results show higher e_50_ in MHAS relative to estimates in LAMBdA (about 3.8 years among women and 3.3 years among men) and similar values relative to WHO.

Table E1. Estimates of life expectancy at age 50 by sex for MHAS 2001-2015 using a Gompertz proportional hazards model, and comparisons with other sources

|  | Women | Men |
| --- | --- | --- |
| MHAS 2001-15 | 30.5 | 28.1 |
| LAMBdA 2005 | 26.7 | 24.8 |
| WHO 2005 | 31.0 | 27.8 |
| WHO 2010 | 31.3 | 28.1 |

**Appendix F. Hazard ratios from Gompertz survival models for the MHAS sample 2001-2015, including interaction terms between age and education and between age and net worth**

TOTAL POPULATION

*p < .05; **p < .01; ***p < .001. AIC denotes Akaike Information Criterion and BIC denotes Bayesian Information Criterion.

WOMEN

*p < .05; **p < .01; ***p < .001. AIC denotes Akaike Information Criterion and BIC denotes Bayesian Information Criterion MEN

*p < .05; **p < .01; ***p < .001. AIC denotes Akaike Information Criterion and BIC denotes Bayesian Information Criterion.

**Appendix G. Sensitivity analysis**

**Hazard ratios from Gompertz survival models for the MHAS sample 2001-2015 for all people aged 50+ and those aged 50-65 at baseline**

WOMEN

MEN
